# The Generalization of Auditory Accommodation to Altered Spectral Cues

**DOI:** 10.1038/s41598-017-11981-9

**Published:** 2017-09-14

**Authors:** Christopher J. G. Watson, Simon Carlile, Heather Kelly, Kapilesh Balachandar

**Affiliations:** School of Medical Sciences, University of Sydney, Sydney, New South Wales 2006 Australia

## Abstract

The capacity of healthy adult listeners to accommodate to altered spectral cues to the source locations of broadband sounds has now been well documented. In recent years we have demonstrated that the degree and speed of accommodation are improved by using an integrated sensory-motor training protocol under anechoic conditions. Here we demonstrate that the learning which underpins the localization performance gains during the accommodation process using anechoic broadband training stimuli generalize to environmentally relevant scenarios. As previously, alterations to monaural spectral cues were produced by fitting participants with custom-made outer ear molds, worn during waking hours. Following acute degradations in localization performance, participants then underwent daily sensory-motor training to improve localization accuracy using broadband noise stimuli over ten days. Participants not only demonstrated post-training improvements in localization accuracy for broadband noises presented in the same set of positions used during training, but also for stimuli presented in untrained locations, for monosyllabic speech sounds, and for stimuli presented in reverberant conditions. These findings shed further light on the neuroplastic capacity of healthy listeners, and represent the next step in the development of training programs for users of assistive listening devices which degrade localization acuity by distorting or bypassing monaural cues.

## Introduction

Effective localization of sound sources depends in part on the comparison of a sound wave’s amplitude and time of onset at each ear. However, these binaural cues (so-called because they rely on inputs to both ears) are inherently ambiguous because the mathematical differences which arise from these comparisons may correspond to any one of several points along the base of a cone that is centered on the interaural axis^1^. In order to pinpoint a sound source location along these ‘cones-of confusion’, the auditory system must additionally be able to recognize in the waveform a pattern of location-specific spectral filtering that arises from the interaction of the incoming sound with the complex shape of an individual outer ear^2^. These monaural, or spectral, cues, in combination with acoustic impact of each individual’s head, are collectively referred to as the head-related transfer functions (HRTFs), and are critical to our sense of three dimensional auditory space. Almost two decades ago, Hofman *et al*.^3^ demonstrated for the first time that the adult auditory system could adapt to perceptually relevant changes in monaural cues, a necessary precondition for the ongoing recalibration to the changes in HRTFs which arise from changes in the shape of the outer ear over the course of a lifetime^4^.

Several recent studies have investigated plasticity in the adult human auditory system when monaural cues are altered^5–10^. It is known that human subjects are able to accommodate to perturbed monaural spectral cues in one ear only^11^, as well as both ears concurrently^8^. Carlile and Blackman demonstrated accommodation to alterations in both the frontal and posterior hemispheres of space–i.e. inside and outside the visual field^7^. Brief periods of exploratory interaction in virtual audio-visual environments improve localization with non-individualized HRTFs^12, 13^. One study found that training with visual feedback on band limited or spectrally warped stimuli improved localization performance^14^. Recently, our laboratory found a similar improvement when subjects combined exploratory behavior with continuous auditory input when compared to visual input alone^8^. Taken together, these studies suggest that auditory-motor interaction is a main driver of accommodation, and that visual input is not necessary for learning a new map of auditory space. However, these studies did not examine whether the accommodation generalizes to different acoustic conditions.

There is a diversity of findings from previous studies of generalization in auditory learning^15^. Human subjects trained at a set of specific pitches improved frequency discrimination performance at both the training frequencies and at untrained frequencies, but the improvement was greater at the trained pitches^16, 17^. While one study found that frequency discrimination learning in one ear did not generalize to the other ear^18^, others found improved performance in the untrained ear^19, 20^. Training in frequency discrimination with stimuli presented for a specific duration elicited no improvement in performance when the duration was altered^18^.

There is also uncertainty as to whether learning in the auditory domain generalizes for spatial tasks. Butler demonstrated listener’s learned associations with narrowband, monaurally heard stimuli did not generalize to new frequencies^21^. Another study examined localization performance of subjects tested with open ears, ear-plugs and ear-muffs, which represent no change, some change and considerable disruption to monaural cues respectively^22^. Improved performance with training generalized from open ear to ear-plug performance and vice versa, but no generalization was seen for localization with ear-muffs. One explanation for this result is the drastic change in outer ear morphology while wearing ear-muffs when compared to the other two conditions. Localization learning may also be assessed using interaural time difference or interaural level difference discrimination, which are the aforementioned binaural cues. Wright and Fitzgerald found that training with interaural level difference or interaural time difference discrimination with pure tones improved discrimination for those tones, but this did not generalize to other frequencies^23^. In contrast, when a complex waveform was used to train interaural level difference discrimination, subjects demonstrated improvements in making similar discriminations for other, untrained complex sounds^24^. This generalizability was presumably due to the increased availability of spectral information in both the complex training and test stimuli in comparison to pure tones.

In light of the above, it cannot be taken for granted that accommodation to perturbed monaural cues generalizes to different conditions. Previously, our laboratory used a sensory-motor training paradigm to accelerate accommodation to altered spectral cues, using localization performance of broadband noises presented over a closed set of spatial locations under anechoic conditions as our baseline and test metric^8^. In this study, we sought to: reproduce the accommodation we had previously observed using our exploratory audio-visual feedback system which relies on broadband noise signals; examine localization performance of broadband noise stimuli at untrained positions under anechoic conditions; compare this to localization ability in echoic conditions; and finally examine whether this learning generalized to more spectro-temporally dynamic speech stimuli.

## Methods

Seventeen subjects (age: 18 to 56 years, 6 females) took part in this study, six of whom had previous experience with auditory accommodation experiments. The subjects were within 15 dB of normal audiometric hearing (125 Hz-8 kHz) as measured by pure tone audiogram. The experimental protocol was approved by the University of Sydney Human Research Ethics Committee. All methods were performed in accordance with the relevant guidelines and regulations. Written informed consent was obtained from all participants.

### Conchal Molds

Our procedure for making inserts to disrupt each participant’s spectral cues has been described in detail elsewhere^8^. In short, after determining the volume of each pinna, Extrude Wash (SDS Kerr) was used to make a negative impression of the concha and surrounding pinna flange. Silicone inserts (Barnes PLATSIL GEL-10) measuring approximately 40% of that figure were made to fit snugly in each concha. The canal entrance remained free of any obstruction. Participants wore these inserts during all waking hours of the 10-day accommodation period.

### Localization Testing Environments, Stimuli and Performance

Per Carlile and Blackman, subjects completed a familiarization task prior to beginning the experiment until they could reliably indicate perceived sound source location using a nose-pointing method^7^. Head orientation was continuously monitored using a Polhemus Fastrak with six degrees of freedom.

Localization testing took place in two environments. The first was a darkened anechoic chamber (described in full here^25^), within which a sound source (Audience A3 loudspeaker) mounted on a robotic arm may be placed in 1° increments around an imaginary sphere with a radius of 1 m. As in Carlile *et al*.^8^, the center of the subject’s head was aligned with the center of the sphere, with the subject facing forward so that each test stimulus was accurately delivered within a stable coordinate system^8^. Localization targets were presented in randomized order to one of 54 target locations which were evenly distributed around the sphere within ± 40° in elevation relative to the audio-visual horizon.

The second test environment was a darkened 3.4 m × 2.4 m × 2.5 m office-space that was a reverberant environment with the time taken for a signal to drop by 60 dB (RT60) being 520 ms. Rather than a mobile robotic arm, the room contained a semicircular array, also of 1 m radius, on which ten Bose Lifestyle Double Cube speakers were situated at 20° intervals. The array was capable of changes in elevation prior to testing, and was adjusted to the participants’ height. It was fixed, during testing such that the speakers presented stimuli on the subject’s audio-visual horizon, 1 m from the centre of the room. Each block was comprised of 20 evenly distributed and randomly ordered trials. The subject starting orientation was varied such that each block of testing presented stimuli to either their left or their right side. Response orientation was recorded using a head mounted InterSense InertiaCube4 motion tracker with three degrees of freedom. For comparisons between anechoic and echoic conditions, subjects performed localization blocks of 20 trials in both this room and the anechoic chamber that were identical, except that the speaker placement in the anechoic chamber required the movement of the robotic arm.

Broadband noise trials involved short bursts of broadband noise stimuli (150 ms duration, 10 ms cosine ramp, 300 Hz−16 kHz). Sound volume roved over a range of 64.5–76.5 dB in 3 dB intervals.

Speech localization tests incorporated monosyllabic words from the Australian version of the Harvard speech corpus^26^ produced by Best *et al*.^27^, which were spoken by a male vocalist from the National Institute of Dramatic Arts in Sydney. The words varied in duration (418–1005 ms) and digitization was performed at a sampling rate of 80 kHz. Sound volume roved over a range of 64.5–76.5 dBA in 3 dB intervals.

Localization performance was quantified using four metrics outlined by Leong and Carlile^28^. The spherical correlation coefficient provided an overall measure of correspondence between the presented locations and the perceived locations. The front-back error rate demonstrated the number of anterior-posterior hemispheric errors (excluding locations within 25° of the interaural axis). Absolute polar angle error scores were also collected for each trial. The polar angle error was normalized using the cosine of the lateral angle to compensate for variation in the area circumscribed by different cones of confusion. Only the front-back error rate was computed in the echoic room as the loudspeakers were located in the horizontal plane.

### Pre-accommodation, accommodation and post-accommodation testing

Following familiarization with the response registration system, subjects performed three sets of tests: five blocks of 54 trials in the darkened anechoic chamber using broadband noise stimuli; 5 blocks of 54 trials in the anechoic chamber using speech stimuli; and finally ten (five left- and five right-sided) blocks of 20 trials each in both the anechoic chamber and the reverberant room using broadband stimuli. These 20-trial blocks involved horizontal plane stimuli such that the robotic arm in the anechoic chamber reproduced the locations of the loudspeakers in the reverberant room. Subjects repeated these three test-types immediately after inserting the conchal molds to demonstrate the acute effect of the mold on sound localization.

The subsequent 10-day accommodation period involved wearing the molds during all waking hours, with daily training on the same 54 stimulus locations in the anechoic chamber. The testing included audio-visual sensory motor feedback designed to accelerate accommodation to new spectral cues as described previously by our laboratory^8^. Following the initial localization response an auditory stimulus was played at the target location which pulsed at a rate proportional to nose-pointing accuracy. Participants were encouraged to explore by moving their heads and to minimize the pointing error using this audio feedback before registering a corrected response. This movement of the head and body ensured that subjects gained sensory-motor feedback information from a continuum of reference locations.

Five blocks of 54 trials were performed on the first and last day, with four blocks performed on all other days.

After the accommodation period, subjects performed localization tests while wearing molds to assess whether their accelerated learning under anechoic conditions generalized to more environmentally relevant stimuli and listening conditions. To investigate whether learning generalized to untrained stimulus locations, subjects completed five blocks of 54 trials at a new set of locations in the anechoic chamber using broadband stimuli. Subjects also localized five blocks at the original 54 trial locations with speech stimuli in anechoic conditions. Lastly, generalization of learning to reverberant conditions was investigated with ten blocks of 20 trials in the reverberant room using broadband stimuli.

We did not collect acute data for the untrained positions in anechoic conditions using broadband stimuli. In this condition, as the alternate locations were placed at interleaved positions compared to the original test locations there was no reason to expect that the acute localization error should be different to the trained locations^25^. In addition, acute testing with this condition could potentially compromise post-accommodation performance by exposing the locations or nature their distribution.

MATLAB 2013a scripts were used for all statistical analysis. The comparisons used one-tailed paired Wilcoxon signed rank tests as the results were not normally distributed. Based upon previous studies, altered spectral cues were expected to decrease localization performance and training was expected to improve performance^3, 7, 8^. Hence one-tailed tests were chosen. For all tests, the significance criterion was *p* < 0.05.

### Data availability

The datasets generated during the current study are available from the corresponding author on reasonable request.

## Results

### The acute effects of the molds and the effects of training on anechoic broadband localization

**Figure 1:**
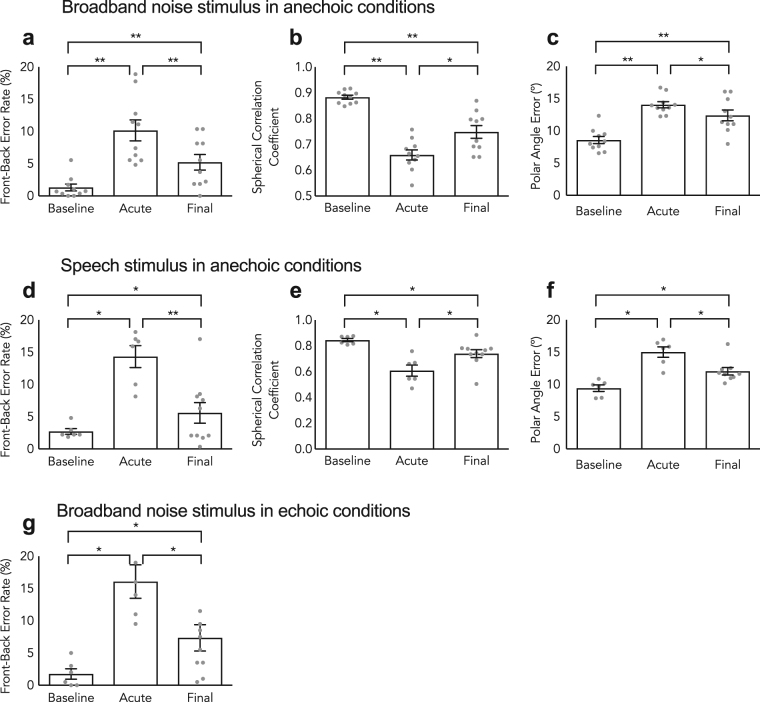
Accommodation to altered spectral cues. Baseline Localization performance with bare ears (i.e. with unaltered spectral cues), immediately after the insertion of molds (i.e. with acutely disrupted spectral cues), with molds at the end of the 10-day training period. Subjects’ performance decreased on acute exposure to molds and improved after the training period. For broadband noise stimuli in anechoic conditions, (**a**) represents the front-back error rate, (**b**) represents the spherical correlation coefficient and (**c**) represents the polar angle error. For speech stimuli in anechoic conditions, (**d**) represents the front-back error rate, (**e**) represents the spherical correlation coefficient and (**f**) represents the polar angle error. For broadband noise stimuli in echoic conditions, (**g**) represents the front-back error rate. Error bars indicate the standard error of the mean. Individual data shown as dots, n = 10. *Denotes p < 0.05, **denotes p < 0.01.

This study first investigated the immediate effect of wearing outer ear inserts on the localization of broadband noise stimuli in anechoic conditions. Figure 1 shows the statistically significant changes between baseline (without mold) and acute (immediately following mold insertion) performance accuracy in terms of front-back error rate (Fig. 1a), spherical correlation coefficient (Fig. 1b) and polar angle error (Fig. 1c) with paired Wilcoxon signed rank tests. The mean front-back error rate increased nearly seven-fold from 1.3% to 10.2% (*p* = 0.001), the mean spherical correlation coefficient decreased by 0.22 from 0.88 to 0.66 (*p* = 0.001) and the mean polar angle error increased from 8.6° to 14.1° (*p* = 0.002). All three metrics indicated a considerable decrease in localization accuracy owing to the distortion of the monaural cues. This result was in accordance with previous findings^7, 8^, although the acute front-back error rate was low relative to studies that restricted bandwidth or warped the spectral cues of stimuli^14^.

We found using paired Wilcoxon signed rank test statistically significant differences between the acute and accommodated rates for front-back confusions, spherical correlation coefficient and polar angle error (Fig. 1). The front-back error rate decreased from 10.2% to 5.2% (*p* = 0.03), the spherical correlation coefficient increased by 0.09 from 0.66 to 0.75 (*p* = 0.01) and the polar angle error decreased from 14.1° to 12.4° (*p* = 0.03). Carlile *et al*.^8^ also described an accelerated improvement in localization performance following the 10-day training period. For every stimulus and for every performance metric, the data show that post-training performance did not reach baseline.

### Acute effects of the molds on speech and reverberant localization

Given the acute impact on broadband stimulus localization performance in anechoic conditions, this study investigated whether a corresponding effect was apparent in more environmentally relevant scenarios. The localization of both speech stimuli under anechoic conditions and broadband stimuli under reverberant conditions for bare ear (baseline) was compared to performance on immediate exposure to the molds (acute) (Fig. 1d,e and f). The group mean front-back error rate for localization of speech stimuli in the anechoic chamber increased more than five-fold from 2.7% to 14.3% upon acute exposure to the molds with a statistically significant difference (Fig. 1d, Wilcoxon signed-rank test, *p* = 0.02). Also, the spherical correlation coefficient metric worsened from 0.84 to 0.61 (Fig. 1e, Wilcoxon signed-rank test *p* = 0.02) and the polar angle error increased from 9.4° to 15° (Fig. 1f, Wilcoxon signed-rank test *p* = 0.03). Similarly, the front-back error rate when localizing broadband noise stimuli in reverberant conditions increased approximately seven-fold from 1.8% to 16.1% with the same level of significance (Fig. 1g, Wilcoxon signed-rank test, *p* = 0.02).

Baseline performance in anechoic and reverberant broadband stimuli had no statistically significant difference in the only measured metric, the front back error rate (*p* = 0.89, Wilcoxon signed rank test). Nor was there any statistically significant difference between baseline performance in anechoic broadband and anechoic speech in front-back error rate (*p* = 0.07, Wilcoxon signed rank test), spherical correlation coefficient (*p* = 0.06, Wilcoxon signed rank test) and polar angle error, (*p* = 0.22, Wilcoxon signed rank test). There was also no difference between acute reverberant broadband front back error rates and acute echoic broadband front back error rates (*p* = 0.09, Wilcoxon signed rank test). Lastly, there was no statistically significant difference in front-back error rate (*p* = 0.18, Wilcoxon signed rank test), spherical correlation coefficient (*p* = 0.49, Wilcoxon signed rank test) and polar angle error, (*p* = 0.43, Wilcoxon signed rank test). Thus the molds had a similar impact on acute performance across all these conditions.

### Generalization of learning to speech localization

The significant decrease in performance in the acute conditions indicated that the distorted spectral cues affected subjects’ localization of speech stimuli under anechoic conditions. While Carlile *et al*.^8^ demonstrated that training with audio-visual sensory motor feedback expedited accommodation to altered spectral cues when localizing the same type of broadband noise stimuli and the same anechoic conditions used during training^8^, this study sought to determine whether equivalent improvements are evident in untrained conditions.

There was a statistically significant improvement in all metrics comparing acute and post-accommodation speech performance. Front-back error rate decreased from 14.7% in acute testing to 5.6% in post-accommodation testing (Fig. 1d, *p* = 0.01, Wilcoxon signed-rank test). The spherical correlation coefficient also improved from 0.61 to 0.74 (Fig. 1e, *p* = 0.02, Wilcoxon signed-rank test). Lastly, the polar angle error decreased from 15.0° to 12.0° (Fig. 1f, *p* = 0.04, Wilcoxon signed-rank test).

**Figure 2:**
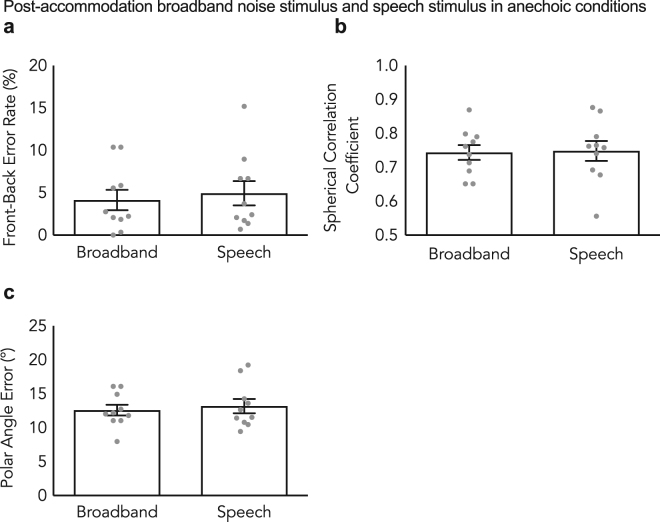
Generalization of learning for speech stimuli in anechoic conditions. Post-accommodation front-back error rate, spherical correlation coefficient and polar angle error in anechoic conditions for both broadband noise stimuli with molds and speech stimuli with molds. (**a**) represents the front-back error rate, (**b**) represents the spherical correlation coefficient and (**c**) represents the polar angle error. Learning generalized to the speech condition. Error bars indicate the standard error of the mean. Individual data shown as dots, n = 10.

Comparisons of post-accommodation performance for both broadband and speech stimuli in the anechoic chamber were also conducted (Fig. 2). There was no statistically significant difference in post-accommodation front-back error rate (*p* = 0.28, Wilcoxon signed-rank test), spherical correlation coefficient (*p* = 0.50, Wilcoxon signed-rank test) or polar angle error (*p* = 0.22, Wilcoxon signed-rank test) for speech and broadband stimuli (Wilcoxon signed-rank test, all *p* > 0.05). This result indicated that the learning facilitated by the sensory-motor interaction with a broadband stimulus did in fact generalize to the more spectrally sparse speech stimuli.

### Generalization of learning to reverberant localization

Previous studies have only done anechoic broadband noise stimulus pre- and post-accommodation testing. In this study we examined whether accommodation also generalized to stimuli under echoic conditions. Front-back error rate decreased from 16.1% in acute testing to 7.2% in post-accommodation testing (Fig. 1g, *p* = 0.01, Wilcoxon signed-rank test). Also, there was no significant difference in post-accommodation front-back error rate for localization of broadband stimuli in anechoic and reverberant conditions (*p* = 0.19, Wilcoxon signed-rank test). Once more this result represented a generalization of learning, in this case from anechoic to echoic conditions.

### Generalization of learning to new stimulus locations

**Figure 3:**
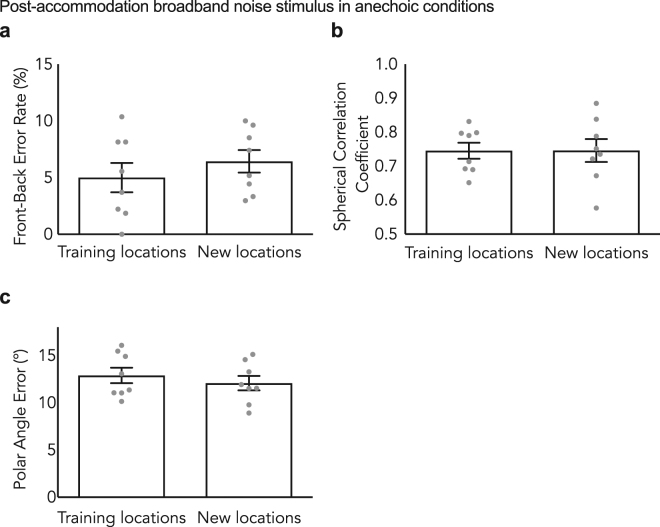
Generalization of learning for untrained positions. Post-accommodation front-back error rate, spherical correlation coefficient and polar angle error in anechoic conditions for broadband noise stimuli with molds presented at both training and new positions. (**a**) represents the front-back error rate, (**b**) represents the spherical correlation coefficient and (**c**) represents the polar angle error. Learning generalized to the speech condition. Error bars indicate the standard error of the mean. Individual data shown as dots, n = 8.

Figure 3 shows 8 participants’ post-accommodation localization accuracy for both the set of locations they were trained on as well as a different, interleaved set of positions. Wilcoxon signed-rank tests found no significant difference in front-back error rate (*p* = 0.08), spherical correlation coefficient (*p* = 0.47) and polar angle error (*p* = 0.23) for the original test and training locations and the new interleaved test locations (Fig. 3). According to these measures, subjects localized the untrained positions with equivalent accuracy to the positions on which they had received daily training, indicating that the accommodation to altered spectral cues generalized to other spatial locations.

## Discussion

This study is novel in demonstrating that accommodation to distorted spectral cues, following training with broadband stimuli in an anechoic environment, generalized to a range of other, more environmentally relevant conditions. As in Carlilie *et al*.^8^, daily training with sensory-motor feedback drove acceleration of learning. Hofman *et al*.^3^ first demonstrated a decrease in localization performance produced by spectral cue distortion using broadband stimuli^3^. We found that this decrease in performance translates to “real-world” stimuli and listening conditions. The reduction of localization performance in both speech and reverberant environments suggests that spectral cue distortion may also present problems for users of hearing aids which distort spectral cues. In general, the localization performance of hearing impaired listeners is worse when using their hearing aids compared to performance unaided and particularly where audibility has been corrected for (for review: see Akeroyd and Whitmer^29^).

The equivalent post-accommodation localization performance of speech and broadband stimuli showed that laboratory training using broadband noise boosted performance in untrained, environmentally relevant conditions. Whitton *et al*.^30^ reported a similar generalization of learning with human subjects trained using localization of broadband noise stimuli and also that subjects exhibited improved localization performance for speech stimuli. Furthermore, laboratory learning generalized to localization in reverberant conditions, with accuracy equivalent to anechoic conditions.

In an area of research largely characterized by uncertainty about auditory learning generalization, the findings of this study stand out in their consistency. One explanation could be that improved learning was afforded by auditory object formation. FMRI studies have suggested that focussing attention on an auditory object results in enhanced neural processing, just as is the case with visual processing^31, 32^. Other studies supported the comparison between the auditory and visual object creation with magneto-encephalography^33^ as well as electro-corticography^34^. A key idea here is that different elements of the stimuli are combined to form an object so that focussing attention on the object can enhance the discrimination of associated attributes^35^. In general, previous studies have focussed on a single localization cue or a single attribute of a stimulus. By contrast, the stimuli used in this study represent the full suite of coherent localization cues which may have enabled a more consistent perceptual learning and generalization. This would support the finding of generalization of interaural time difference and interaural level difference discrimination when trained with complex tones^24^ compared to its absence when trained with simple tones^23^. Another possible explanation is that all the stimulus types used in this study can be localized by a single computational process that is robust to the spectral variations in these stimuli. For instance, models of spectral cue matching that rely on extraction of the spectral gradient are robust to irregularity in source spectra^36, 37^. In the context of the current experiments, as the individual stimuli were presented on a background of silence, grouping and object formation is less relevant and spectral cue matching models might be expected to perform well. Such correlational models are founded on simple, physiologically conceivable process but further work is required to establish whether they are sufficiently robust in the face of the sorts of spectral distortions produced by the conchal molds.

Another relevant finding of this study was that post-accommodation localization accuracy was equivalent for previously unseen sound locations for front-back error, polar angle error and spherical correlation coefficient scores. The accelerated accommodation to spectral cues described in Carlile *et al*.^8^ cannot be explained by memorization of the stimulus positions and the improvements most likely reflect true auditory perceptual learning.

While this study adds to the body of research demonstrating that the adult auditory system is capable of perceptual learning of altered spectral cues^3, 5, 7, 8^, it remains an open question as to whether hearing-impaired individuals are capable of accelerated accommodation to spectral cues. Encouragingly, the weight of evidence suggests that they are capable of functional neuroplasticity^38, 39^. The findings presented here have significant implications for training paradigms for users of hearing assistive devices.
